# A Proposed Microneedle–Small Extracellular Vesicle System for Localized Adjunctive Treatment of Established Oral Squamous Cell Carcinoma: A Narrative Review and Preclinical Development Perspective

**DOI:** 10.3390/cells15171527

**Published:** 2026-08-24

**Authors:** Hai He, Zishuai Chen, Hengxiang Liu, Xiaohua Zhang, Zhiqiang Li, Heqiang Yang, Xiaoyong Chen, Yufei Yang, Zifan Wang

**Affiliations:** 1 Key Laboratory of Biotechnology and Bioengineering of State Ethnic Affairs Commission, Biomedical Research Center, School of Bioengineering, Northwest Minzu University, No. 1 Xibeixincun, Baiyin Road, Chengguan District, Lanzhou 730030, China; haihe1013@outlook.com (H.H.); czs2410188708@outlook.com (Z.C.); 18324841356@163.com (H.L.); heqiang.yang@outlook.com (H.Y.); xiaoyongchen678@gmail.com (X.C.); qruipharm@163.com (Y.Y.); 2 Gansu Animal Cell Technology Innovation Center, Northwest Minzu University, No. 1 Xibeixincun, Baiyin Road, Chengguan District, Lanzhou 730030, China; 3 Key Laboratory of Oral Diseases of Gansu Province, Key Laboratory of Stomatology of State Ethnic Affairs Commission, School of Stomatology, Northwest Minzu University, No. 1 Xibeixincun, Baiyin Road, Chengguan District, Lanzhou 730030, China; 17393107697@163.com (X.Z.); lizhiqiang6767@163.com (Z.L.)

**Keywords:** oral squamous cell carcinoma, microneedle, small extracellular vesicles, localized drug delivery, preclinical development

## Abstract

Established oral squamous cell carcinoma (OSCC) remains constrained by local recurrence, inadequate lesion exposure, systemic toxicity, and therapy resistance. This narrative review evaluates a proposed microneedle–small extracellular vesicle (sEV) system for localized adjunctive treatment in three preclinical contexts: postoperative residual-disease control, local immunomodulation/checkpoint combination, and chemoradiotherapy sensitization. Direct evidence for the combined microneedle–sEV system in OSCC is not yet available; the analysis therefore integrates OSCC-specific sEV mechanisms with oral-mucosal microneedle and cross-disease engineering studies. We focus on whether the proposed microneedle–sEV system can preserve sEV potency, achieve reproducible local delivery, and meet oncologic-safety, manufacturing, and repeated-dose oral-safety requirements. The review provides a preclinical decision framework and comparator-based development criteria rather than a claim of clinical readiness.

## 1. Introduction

Oral squamous cell carcinoma (OSCC) accounts for more than 90% of oral malignancies and caused approximately 188,000 deaths worldwide in 2022 [1,2]. Survival remains substantially worse in locally advanced, recurrent, or metastatic disease, while treatment can impair speech, swallowing, appearance, and psychosocial function [3,4,5,6].

Current OSCC management combines surgery, chemoradiotherapy, targeted therapy, and immunotherapy [7]. Persistent problems include positive or close margins, local recurrence, inadequate intratumoral exposure, systemic toxicity, resistance, and limited response to PD-1/PD-L1 monotherapy [8,9,10,11,12,13,14,15], motivating more precise local-delivery approaches.

Small extracellular vesicles (sEVs) can transport nucleic acids, proteins, and drugs [16,17,18]. Consistent with MISEV2023, “sEVs” is used for small EV preparations without demonstrated endosomal origin; “EVs” is reserved for broader or size-unspecified populations. “Exosome” or “exosomal” is retained only in exact search terms, reference titles, or when an endosomal biogenetic origin has been specifically demonstrated [19]. For grammatical consistency, “sEV” is used attributively (e.g., sEV cargo, sEV potency, and sEV loading), whereas “sEVs” is used when referring to vesicles as a plural noun.

System rationale: sEVs are candidate biological carriers, but their properties depend on source and preparation. Free sEVs face salivary clearance, enzymatic degradation, and limited penetration across stratified oral epithelium [20,21,22,23], whereas oral-mucosal microneedles can bypass this barrier and support local delivery [24]. Relevant evidence therefore comes from OSCC sEV biology, oral microneedle studies, and combined microneedle–sEV systems in other diseases [25,26,27,28].

This review is restricted to a proposed microneedle–sEV system for established OSCC in three preclinical contexts: postoperative residual-disease control, local immunomodulation/checkpoint combination, and chemoradiotherapy sensitization. OPMD prevention and stand-alone regenerative applications are excluded. Diagnostic/theranostic applications remain outside the core therapeutic analysis and are considered only briefly as a future extension in Section 5.4. The central question is whether a candidate microneedle–sEV system can deliver a potent local sEV dose and show added value over appropriate local comparators without unacceptable mucosal or oncologic risk.

Search strategy: PubMed and Web of Science Core Collection were searched on 31 March 2026 for the 2019–2025 literature using (“exosome” OR “small extracellular vesicle”) AND (“microneedle” OR “micro-needle”) AND (“oral cancer” OR “oral squamous cell carcinoma” OR “OSCC”), supplemented by citation chaining and selected foundational or online-first studies.

Eligibility and evidence mapping: Experimental, translational, clinical, methodological, and regulatory studies relevant to the three focused contexts or their component technologies were considered. Diagnostic-only, OPMD-only, and unrelated regenerative studies were excluded from the core therapeutic synthesis, except for the limited salivary ncRNA literature used to contextualize the future diagnostic extension in Section 5.4; oral-wound data were retained only when they informed delivery or safety. We used an author-defined five-level hierarchy based on disease specificity and system completeness; it indicates experimental proximity, not methodological quality.

**Figure 1:**
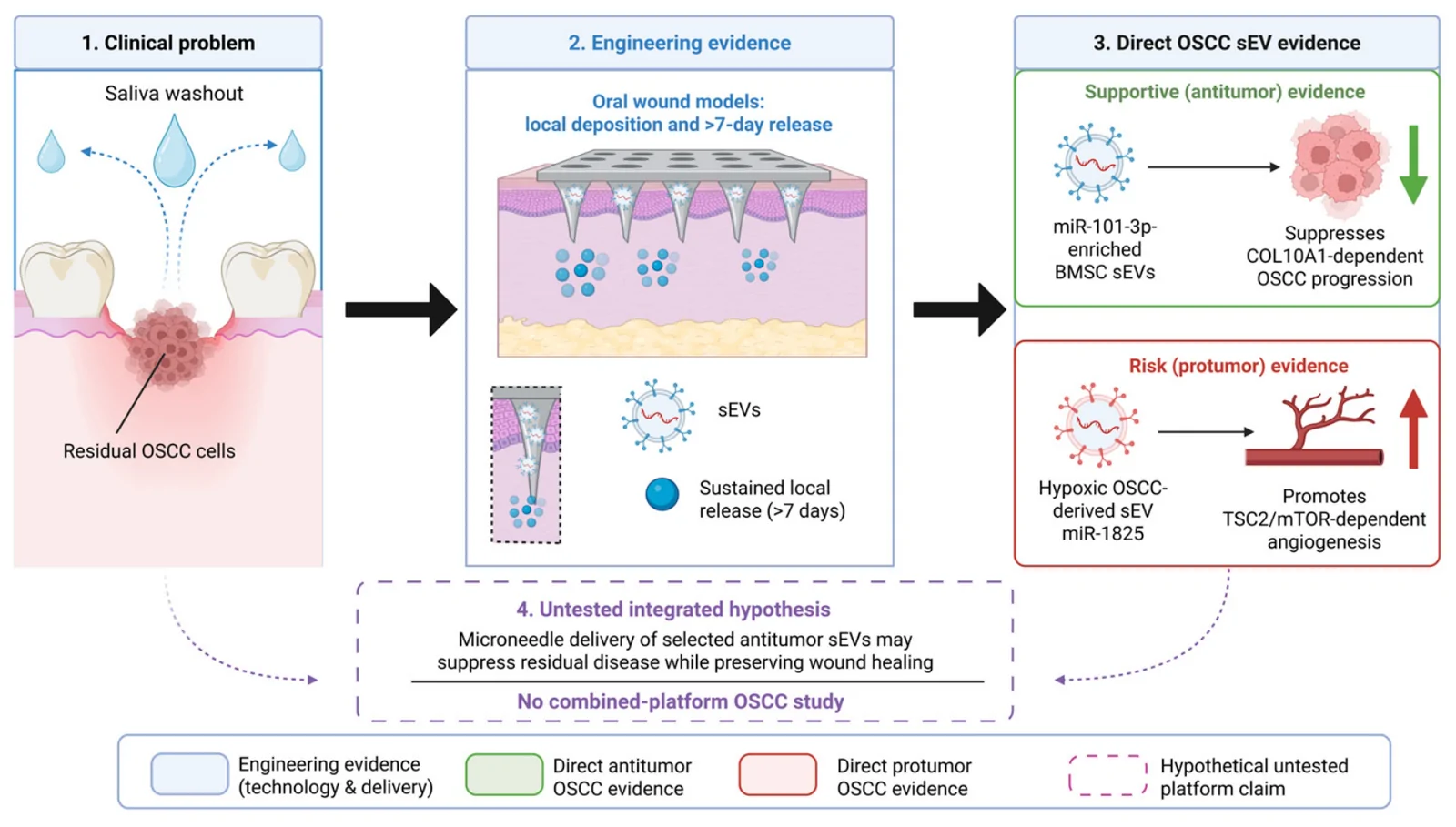
Evidence-qualified conceptual model of a proposed postoperative microneedle–sEV system in established OSCC. Direct OSCC studies support antitumor miR-101-3p/COL10A1 signaling and identify proangiogenic miR-1825/TSC2–mTOR risk [82,98]; oral studies support mucosal adhesion, deposition, and sustained sEV release [20,21,22,23,103,104]. The integrated postoperative outcome remains conceptual.

**Figure 2:**
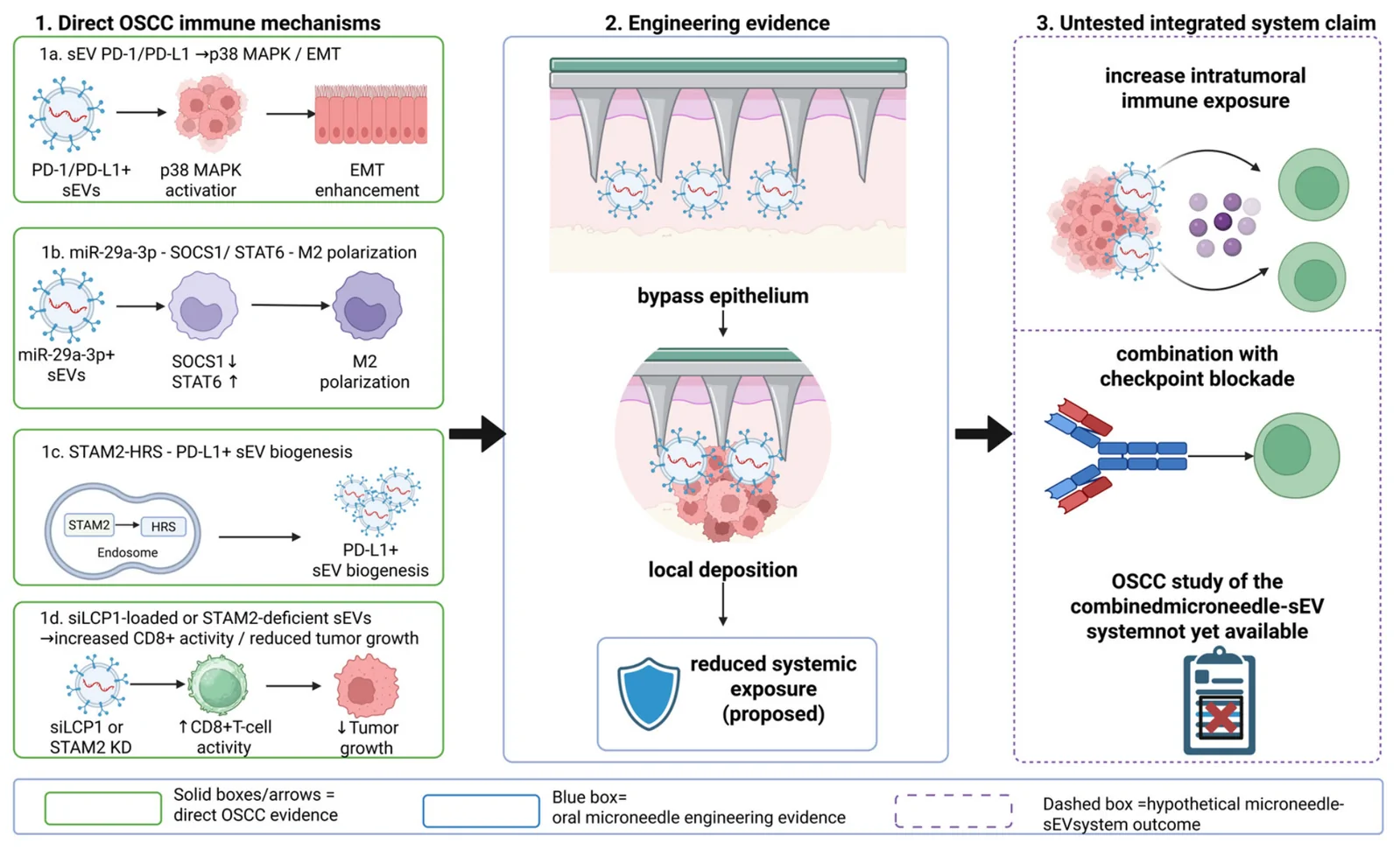
Evidence-qualified conceptual model of a proposed microneedle–sEV system for local immunomodulation in established OSCC. Direct OSCC evidence includes PD-1/PD-L1–p38 MAPK, miR-29a-3p/SOCS1–STAT6, STAM2–HRS/PD-L1, siLCP1/LCP1, and CD8+ T-cell effects [83,84,88,89,99]; microneedle studies support local delivery only [103,119,120].

**Figure 3:**
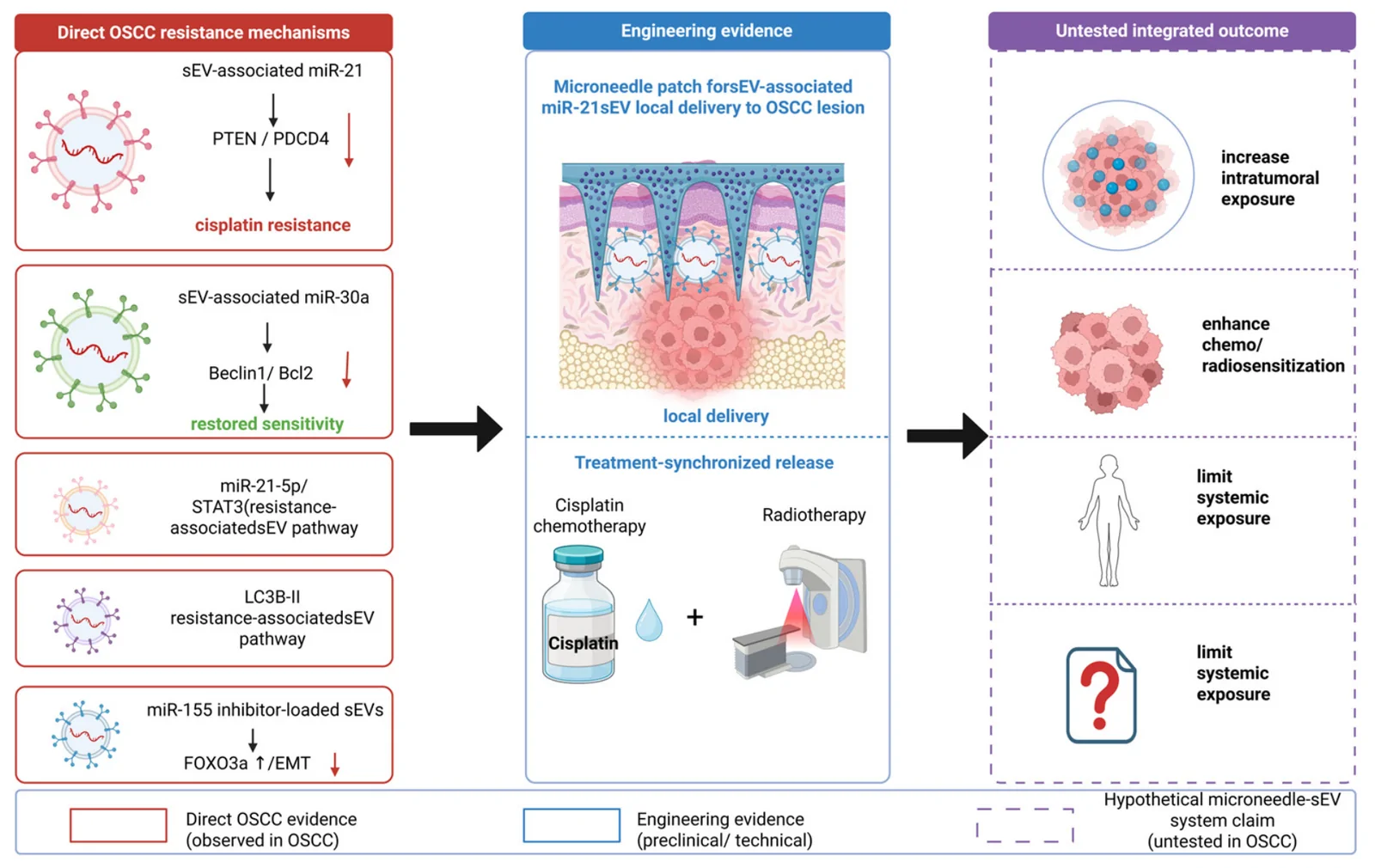
Evidence-qualified conceptual model of a proposed microneedle–sEV system to chemoradiotherapy sensitization in established OSCC. Direct OSCC evidence includes miR-21/PTEN–PDCD4, miR-30a/Beclin1–Bcl2, miR-21-5p/STAT3, LC3B-II, and miR-155/FOXO3a–EMT pathways [86,87,90,91,92]; non-OSCC studies support local delivery and treatment-synchronized release as engineering possibilities [28,124,125,126,127]. Red arrows indicate activating or promoting effects, while red and green text denote adverse/pro-tumor and beneficial/therapeutic effects, respectively.

**Figure 4:**
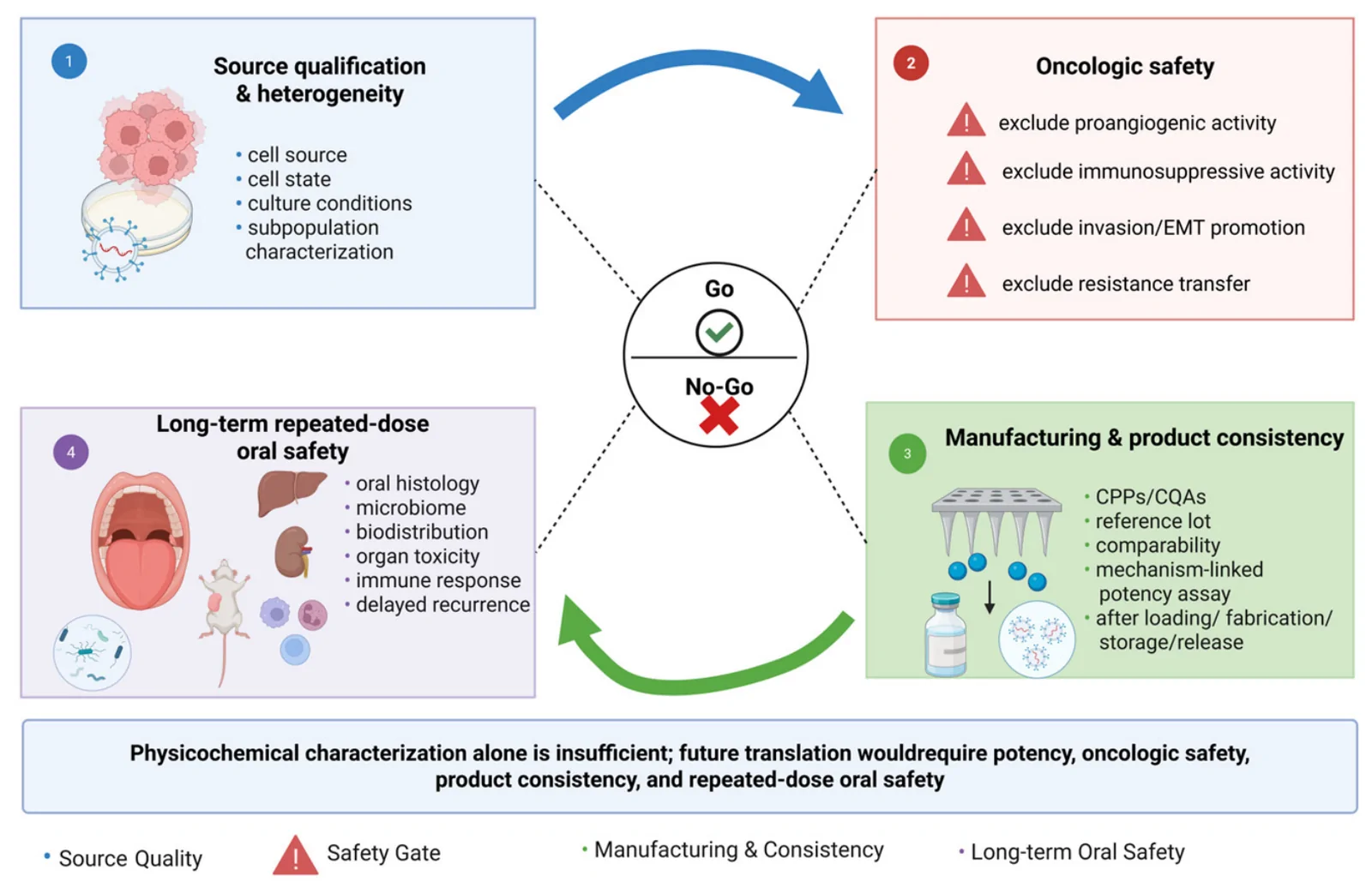
Consolidated preclinical development gates for a proposed microneedle–sEV system in OSCC. Progress depends on source qualification/oncologic safety, reproducible manufacturing with mechanism-linked potency, defined in vivo fate, and repeated-dose oral safety [19,82,84,85,86,88,89,136,139,158,159,160,161,162,163,164].

Evidence-qualified wording is used throughout: “reported/demonstrated” denotes findings tested in the cited model, “supports feasibility” denotes component-level evidence, and “may/could/proposed/conceptual” denotes extrapolation. The integrated construct is termed a “microneedle–sEV system” throughout. When referring to the OSCC concept rather than a directly tested formulation, this term is consistently qualified as proposed, candidate, or conceptual. Figure 1, Figure 2, Figure 3 and Figure 4 use the same terminology and evidence distinction.

## 2. Clinical Rationale for the Proposed Microneedle–sEV System

### 2.1. Comparison with Alternative Local Delivery Platforms

Table 1 summarizes the principal clinical and local-delivery constraints. Hydrogels and mucoadhesive films can prolong superficial residence but have limited epithelial penetration; microneedles improve mucosal access but must retain strength and adhesion in the wet, mobile oral environment [29,30,31,32,33,34,35,36,37,38]. Injectable depots provide prolonged release but are invasive and difficult to repeat, whereas nanoparticles can improve solubility yet remain vulnerable to salivary clearance and variable mucosal penetration [30,38,39,40,41,42].

PRV111 provides a clinically relevant local comparator: In a phase I/II study of early-stage oral cavity squamous cell carcinoma, neoadjuvant PRV111 met its prespecified efficacy threshold of ≥30% tumor-volume reduction and produced a mean tumor-volume reduction of 69% over approximately 7 days, with a response rate above 87% [38]. The platform remains a cisplatin-based local delivery system rather than a biological-cargo platform. Synthetic carriers offer easier scale-up and programmable release, whereas sEVs offer biological cargo but introduce source, loading, and consistency challenges [41,43,44,45,46,47,48,49,50,51,52]. The proposed microneedle–sEV system is therefore a testable combination of epithelial access and biological cargo, not an established superior alternative.

### 2.2. Limitations of Current OSCC Treatment

The treatment-related constraints most relevant to local adjunctive delivery are summarized in Table 1. Surgery can leave positive or close margins and substantial local-recurrence risk [53,54,55,56]; cisplatin-based chemoradiotherapy and cetuximab are limited by heterogeneous tumor exposure, resistance, and dose-limiting toxicity [57,58,59,60,61]; and PD-1/PD-L1 monotherapy benefits only a minority of patients in an immunosuppressive OSCC microenvironment [62,63,64,65].

### 2.3. Advantages and Limitations of Local Administration

Because many OSCC lesions and surgical beds are accessible, local administration may increase lesion exposure while reducing systemic dose [38,66,67]. Existing options involves trade-offs in invasiveness, epithelial penetration, retention, and repeatability: Injections diffuse rapidly and are difficult to repeat; patches/hydrogels remain mainly superficial; and implantable depots require surgery [38,66,67,68,69,70,71,72,73,74,75,76,77,78,79,80]. A candidate local adjunct should therefore be judged on epithelial penetration, wet adhesion, controlled release, repeatability, and mucosal safety rather than on local delivery alone.

**Table 1:** Key challenges in mainstream clinical treatment regimens for OSCC and limitations of local delivery methods.

Level of Care	Method	Key Limitations	Representative Outcomes	Main Cause	Refs.
Systemic treatment	Curative surgery	Local recurrence risk	Positive margins: 10–25% Local recurrence: 30–40%	Limited resection margins No effective postoperative local therapy	[54]
	Concurrent chemoradiotherapy (cisplatin)	Systemic toxicity Insufficient lesion exposure	Grade ≥3 oral mucositis: 40–60% Bone marrow suppression: 20–30%	Physiological barriers Low local exposure	[60,61]
	Targeted therapy (cetuximab)	Restricted eligible population Acquired resistance	Effective only in selected molecular subgroups	Single-target mechanism Limited resistance control	[12,13]
	Immunotherapy (PD-1/PD-L1 inhibitors)	Low ORR Immune-related adverse events	Pembrolizumab monotherapy ORR: 16.9% (total R/M HNSCC)	Immunosuppressive tumor microenvironment Systemic immune activation	[62]
Topical treatment	Intratumoral injection	Invasive administration Rapid drug diffusion	Retention: only a few hours Repeated long-term dosing impractical	Poor compliance Infection risk	[67,68]
	Patches/hydrogels	Limited mucosal penetration	Reported macromolecule penetration: <1% Reported retention: 1–2 h	Very low bioavailability Subtherapeutic local concentration	[38,69,70]
	Intratumoral formulation	Surgical implantation Limited dose control	Restricted to surgical settings	Unsuitable for non-surgical patients Limited dosing flexibility	[80]

## 3. Proposed Microneedle–sEV System for Established OSCC

Oral-mucosal microneedle studies support access and retention, whereas OSCC sEV studies provide biological mechanisms [81]. The following sections therefore separate direct OSCC evidence from microneedle engineering evidence across three proposed applications, with the shared evidence boundary and decision criteria consolidated in Section 3.5 and Table 2.

### 3.1. OSCC-Specific Mechanistic Foundation

Before considering the proposed microneedle–sEV system, the biological premise should be anchored in OSCC-specific sEV mechanisms rather than cross-disease analogy. Direct studies implicate sEV signaling in angiogenesis/metastatic plasticity, immune escape, and cisplatin response [82,83,84,85,86,87]. Under hypoxia, OSCC-derived sEV miR-1825 increased endothelial proliferation, migration, invasion, and tube formation through TSC2/mTOR, whereas miR-1825 inhibition reversed these effects [82]. Circulating sEV PD-1 also engaged tumor PD-L1 and activated p38 MAPK-associated senescence/EMT, and higher circulating sEV PD-1 correlated with lymph-node metastasis [83].

Immune effects are also OSCC-specific. OSCC sEV miR-29a-3p targeted SOCS1 in macrophages, increased STAT6 activation and M2 polarization, and enhanced OSCC-cell proliferation/invasion; restoring SOCS1 counteracted this effect [84]. EV-associated mtDNA D-loop/PD-L1 was linked to regulatory T-cell activity and adverse clinicopathological features [85], while M2-macrophage sEV miR-23a-3p/PTEN signaling and STAM2–HRS-dependent PD-L1-positive sEV biogenesis provide additional tumor-promoting or immune-evasion mechanisms [88,89].

For cisplatin response, resistant-cell sEV miR-21 transferred resistance through PTEN/PDCD4 suppression, whereas sEV-mediated miR-30a reduced Beclin1/Bcl2 and restored cisplatin sensitivity [86,87]. Additional OSCC component studies implicate miR-21-5p/STAT3, LC3B-II, and miR-155 inhibitor/FOXO3a–EMT pathways [90,91,92]. These mechanisms are retained because they provide disease-specific cargo and pharmacodynamic anchors, but they represent distinct sEV products rather than one unified formulation.

### 3.2. Proposed Application: Postoperative Residual-Disease Control

Postoperative local recurrence remains a major treatment problem; the 2-year rate after radical surgery for early/intermediate disease is nearly 30% [93,94,95]. A proposed local adjunct would need to suppress residual OSCC while preserving wound healing and avoiding the burden of repeated injections [67,68,93].

Antitumor component evidence: Menstrual mesenchymal stem cell-derived sEVs inhibited angiogenesis, miR-101-3p-enriched BMSC sEVs suppressed COL10A1-dependent OSCC proliferation/invasion, and siLCP1-loaded sEVs reduced OSCC progression in separate models [96,97,98,99].

Repair evidence and biological caution: Plant-derived EV-like nanovesicles and stem-cell sEVs promoted epithelial repair, collagen organization, angiogenesis, or inflammation resolution in separate wound models [100,101]. These observations are relevant to postoperative tolerability, but repair-associated signaling could also stimulate residual malignant cells [102]. The concern is reinforced by direct OSCC evidence that hypoxia-induced sEV miR-1825 activates proangiogenic TSC2/mTOR signaling [82]. Thus, postoperative development should couple wound-healing endpoints with residual-tumor and pathway-level safety measurements rather than assuming that regenerative activity is uniformly beneficial.

Oral-ulcer microneedle studies support wet adhesion to irregular mucosa, epithelial penetration, sustained sEV release for more than 7 days, and reduced local inflammation [27,103,104]. These Level 2 data are useful for postoperative delivery design, but the relevant OSCC experiment must evaluate residual-tumor control, wound healing, local retention, systemic biodistribution, and oncologic safety in the same model (Figure 1).

### 3.3. Proposed Application: Local Immunomodulation and Checkpoint Combination

Direct OSCC mechanisms: sEV PD-1/PD-L1–p38 MAPK signaling, miR-29a-3p/SOCS1–STAT6-driven M2 polarization, and STAM2–HRS-dependent PD-L1-positive sEV biogenesis provide direct mechanistic anchors for local immunomodulation [83,84,89].

Immune-context implication: OSCC contains suppressive myeloid cells, M2 macrophages, regulatory T cells, and relatively limited cytotoxic T-cell activity [105,106,107]. Tumor-, macrophage-, and hypoxia-associated sEVs can influence T-cell inhibition, macrophage polarization, migration, angiogenesis, chemosensitivity, and EMT/invasion [88,108,109,110,111]. Consistent with these OSCC data, broader studies have established roles for exosomes in cancer progression, tumor–microenvironment communication, immunosuppression (including NK-cell modulation), and anticancer therapy [112,113,114,115,116]. This source-dependent biology supports local immunomodulation as a testable concept but also makes source qualification and cell-specific target engagement essential.

In KEYNOTE-048, pembrolizumab monotherapy achieved an objective response rate of 16.9% in the total recurrent/metastatic HNSCC population [62]. Immune-related adverse events and OSCC-specific combination strategies remain clinically relevant considerations [117,118]. In OSCC models, EBI3-displaying siLCP1-loaded sEVs reduced LCP1 expression and tumor growth, whereas STAM2-deficient OSCC sEVs increased proliferating and granzyme B-positive CD8+ T cells [89,99]. These findings support candidate immune mechanisms, not an established microneedle/checkpoint combination.

Studies of microneedle–sEV systems in oral ulcers and superficial non-oral tumors inform local immune delivery, macrophage modulation, and reduced systemic exposure as engineering possibilities [28,103,119,120,121]. However, reparative M2 polarization in ulcer models should not be equated with antitumor immunity because M2-like macrophages and their sEV cargos may support OSCC progression [88,105,106,107]. Figure 2 therefore separates the OSCC immune mechanisms from the delivery evidence and the hypothetical checkpoint-combination outcome.

### 3.4. Proposed Application: Chemoradiotherapy Sensitization

Cisplatin-based chemoradiotherapy is limited by inadequate tumor exposure and acquired resistance [10,122,123]. The proposed role of microneedles is therefore local delivery of sensitizing sEV cargos rather than replacement of standard therapy.

Direct OSCC studies show resistance transfer through sEV miR-21/PTEN–PDCD4 and resensitization through sEV miR-30a/Beclin1–Bcl2 [86,87]. Additional component studies implicate miR-21-5p/STAT3, LC3B-II, and miR-155 inhibitor/FOXO3a–EMT pathways [90,91,92]; these represent distinct candidate mechanisms, not one unified sEV product.

Non-OSCC studies of microneedle–sEV systems support local deposition, controlled release, and antitumor-delivery feasibility [28,124]. For oral translation, salivary washout, mucosal movement, epithelial structure, and OSCC stroma remain relevant engineering constraints. Preclinical OSCC testing should therefore compare the proposed microneedle–sEV system with free sEVs, blank microneedles, standard chemoradiotherapy, and an established local comparator while measuring sensitization magnitude, intratumoral exposure, synchronized release, mucosal injury, systemic exposure, and repeated-dose safety (Figure 3).

### 3.5. Cross-Cutting Evidence Boundary and Decision Criteria

Across all three contexts, OSCC-specific sEV biology and microneedle delivery have largely been studied separately. We therefore use one cross-cutting decision framework: preservation of sEV identity/potency, reproducible local dose, limited systemic escape, OSCC-specific target engagement, and added value over free sEVs, blank microneedles, and an established local comparator.

**Table 2:** Focused evidence map for a proposed localized microneedle–sEV system in established OSCC.

**Part A: OSCC-Specific Mechanistic and sEV Evidence (Level 3; No Combined Microneedle Delivery Tested)**					
**Context**	**Reported sEV Finding**	**MN Role/Boundary**	**Refs.**	**Level**	**Next Test**
OSCC mechanism: angiogenesis/metastasis	miR-1825 → TSC2/mTOR angiogenesis; sEV PD-1 → PD-L1/p38-MAPK EMT and nodal-metastasis association	OSCC mechanism only; MN delivery not tested	[82,83]	Level 3	Exclude proangiogenic/prometastatic activity
OSCC mechanism: immune escape	miR-29a-3p → SOCS1/STAT6 M2 polarization; mtDNA/PD-L1 → Treg activity; STAM2–HRS → PD-L1-positive sEV biogenesis	OSCC mechanism only; non-OSCC MN data inform engineering	[84,85,89]	Level 3	Confirm target engagement and immune-cell effects in OSCC
OSCC mechanism: cisplatin response	miR-21 → PTEN/PDCD4 suppression and resistance; miR-30a → Beclin1/Bcl2 suppression and resensitization	OSCC mechanism only; MN contribution untested	[86,87]	Level 3	Confirm cargo delivery, pathway modulation, and causal rescue
Postoperative residual disease	Menstrual MSC-derived sEVs: anti-angiogenic and tumor-suppressive effects	Oral studies support wet adhesion and prolonged local delivery	[97,103,104]	Level 3	Test residual-tumor control + wound safety
Postoperative residual disease	miR-101-3p-enriched BMSC sEVs → COL10A1-dependent suppression of proliferation/invasion	Controlled surgical-bed release proposed	[98,103,104]	Level 3	Verify post-fabrication cargo potency
Local immunomodulation	EBI3-displaying, siLCP1-loaded sEVs → lower LCP1 and OSCC progression	Localized immune delivery proposed	[99]	Level 3	Compare free sEVs, the microneedle–sEV system, and checkpoint blockade
Local immunomodulation	STAM2-deficient OSCC sEVs → more proliferating/GzmB-positive CD8+ T cells	Distinct product; local MN delivery untested	[89]	Level 3	Define source, potency, and immune-cell uptake
Chemoradiotherapy sensitization	Ovatodiolide-associated sEV modulation → lower miR-21-5p/STAT3 signaling	Treatment-synchronized delivery proposed	[90]	Level 3	Test added sensitization vs. standard therapy
Chemoradiotherapy sensitization	miR-155 inhibitor-loaded sEVs → FOXO3a↑, EMT↓, cisplatin sensitivity↑	OSCC spheroid/xenograft evidence; MN delivery untested	[92]	Level 3	Verify potency after loading and synchronized release
**Part B: Oral-Delivery and Cross-Disease Engineering Evidence (Levels 2, 4, and 5; Not Used as OSCC Mechanistic Evidence)**					
**Context**	**Reported sEV finding**	**MN role/boundary**	**Refs.**	**Level**	**Next test**
Postoperative delivery	Oral-ulcer microneedle–sEV systems → improved healing and reduced inflammation	Oral adhesion/local delivery; not residual OSCC	[103,104]	Level 2	Guide delivery and wound-safety design
Local immunomodulation	Engineered sEVs in superficial non-oral tumors → enhanced local antitumor immunity	Microneedle–sEV system evidence outside OSCC	[28,120]	Level 5	Use as engineering evidence only
Chemoradiotherapy sensitization	Engineered sEVs in non-oral tumors → improved local delivery/antitumor response	Microneedle–sEV system delivery outside OSCC	[28,124]	Level 5	Validate in orthotopic OSCC
Cross-cutting oral delivery	MN without sEV cargo → oral-mucosal penetration	Device-only oral evidence	[26,128]	Level 4	Define site-specific insertion and safety

Author-defined evidence levels: Level 1, testing of the combined microneedle–sEV system in OSCC; Level 2, testing of microneedle–sEV systems in oral mucosal disease; Level 3, sEV-only OSCC evidence; Level 4, oral microneedle-only evidence; and Level 5, microneedle–sEV system evidence from non-OSCC models. The hierarchy reflects experimental proximity, not methodological quality; non-OSCC evidence is used only for engineering questions.

## 4. Candidate Technical Design of the Proposed Microneedle–sEV System

Candidate design is discussed only where it affects the three proposed OSCC applications.

### 4.1. Matrix Materials and Microneedle Structure

Oral microneedles must retain mechanical integrity and adhesion under saliva, movement, microbiota, and irregular mucosal geometry [103,119]. Hyaluronic acid offers biocompatibility and swelling but limited strength; chitosan provides mucoadhesion and antibacterial activity but lower solubility; and silk fibroin provides strength and controlled degradation but often needs adhesive blending or surface modification [128,129,130,131,132,133]. Composite matrices may balance these properties, although no formulation is established as optimal for OSCC.

Needle geometry should be site-specific because oral mucosal thickness, movement, and neurovascular anatomy vary by location. Oral studies have evaluated needle lengths of roughly 500–1000 μm, but insertion depth, array density, fracture resistance, and backing design remain device- and site-dependent [26,128,134,135]. Mechanical strength, adhesion, insertion, and release should therefore be tested in relevant ex vivo tissues under simulated salivary flow before in vivo validation [36,134,136,137,138,139,140].

### 4.2. sEV Loading and Bioactivity Preservation

Matrix embedding, physical adsorption, and chemical immobilization offer different trade-offs between loading, retention, and processing stress [27,141,142,143,144,145]. Embedding can provide high and uniform loading but may produce burst release or drying damage; adsorption is mild but has lower loading and weaker binding; and covalent/chemical immobilization can improve retention while risking alteration of sEV surface proteins. Selection should therefore be driven by dose, release duration, and acceptable processing stress.

Bioactivity preservation favors mild, low-temperature fabrication, low-toxicity crosslinking, stabilizers such as trehalose or mannitol, and aseptic processing [27,146]. HA/trehalose microneedles have preserved sEV morphology, particle size, CD63 expression, and functional activity in component studies [147,148]. Therapeutic proteins have likewise been loaded into microneedles for local delivery [149]. These observations support the feasibility of protective matrices, but each candidate OSCC product still requires direct testing of sEV identity, membrane integrity, cargo retention, and mechanism-linked potency after fabrication, sterilization, storage, and release.

### 4.3. Release Kinetics and Targeting

Release and targeting should be application-specific [150]. Matrix degradation, polymer molecular weight, crystallinity, and crosslink density can tune release. Sustained exposure may be tested for postoperative residual-disease control, whereas treatment-synchronized or lesion-responsive profiles may be explored for immunotherapy or chemoradiotherapy combinations [125,126,127,151]. These are candidate design strategies derived from component studies rather than established OSCC dosing requirements.

Physical targeting can exploit direct visual placement over accessible lesions and surgical beds. Molecular targeting through EGFR- or integrin-binding ligands may further increase tumor-cell uptake, but it adds formulation complexity and must preserve sEV function, off-target safety, and manufacturing reproducibility [152,153]. The candidate technical design options discussed above are summarized in Table 3.

### 4.4. Preclinical In Vitro and In Vivo Evaluation

In vitro evaluation: Measure release under simulated oral conditions, mucosal penetration/retention, cellular uptake, cytotoxicity, sensitization, and immunomodulation; patient-derived OSCC organoids can supplement conventional models [26,120,128,153,155,156].

In vivo evaluation: Prioritize orthotopic and post-resection recurrence models and quantify local retention, biodistribution, tumor control, immune effects, treatment response, mucosal injury, systemic exposure, and repeated-dose safety [157].

Mechanistic validation: Predefine uptake, cargo delivery, target engagement, downstream pathway modulation, and rescue/abrogation experiments [82,84,86,87]. Tumor response without corresponding target engagement should not be interpreted as validation of the proposed sEV mechanism.

We propose four preliminary go/no-go criteria for the proposed microneedle–sEV system:

- Preserve sEV identity and mechanism-linked potency after fabrication.
- Deliver a reproducible local dose with adequate retention and limited systemic escape.
- Show added value over free sEVs, blank microneedles, and an established local comparator.
- Meet context-specific efficacy and oral-safety requirements.

Failure of these criteria should halt or redesign the candidate formulation.

## 5. Consolidated Preclinical Development Barriers

The recurring development issues—sEV heterogeneity/pro-tumor risk, in vivo fate, product consistency, repeated-dose safety, and scale-up—are consolidated here and summarized in Figure 4 and Table 4 rather than repeated across application sections.

### 5.1. sEV Heterogeneity and Oncologic Safety

sEV composition and potency vary with producer-cell identity/state, culture conditions, isolation or enrichment, and downstream processing [19,158,159,165,166,167,168,169,170,171,172,173,174,175,176,177,178,179,180]. This variability affects cargo, purity, potency, and batch comparability. Direct OSCC studies further show that sEVs can transmit proangiogenic, immunosuppressive, invasive/EMT, or treatment-resistance signals [82,84,85,86,88,89]. Source qualification should therefore combine cell-bank/source controls, identity/purity measures, mechanism-linked potency, and negative oncologic-safety testing rather than relying on particle markers alone.

Preparations showing reproducible proangiogenic, pro-invasive, immunosuppressive, or resistance-transferring activity should not advance unless engineering neutralizes the relevant risk [82,84,85,86,88,158,159].

### 5.2. In Vivo Fate and Repeated-Dose Oral Safety

Key in vivo uncertainties are oral-tumor penetration, retention, cellular uptake, systemic escape, clearance, and repeated-dose toxicity. Fluorescent, bioluminescent, or radiotracer approaches can quantify biodistribution and should be linked where possible to spatial or molecular readouts of biological effect [181,182,183]. Existing oral-mucosal insertion studies, short-term skin data, and route-specific EV toxicology provide only partial safety support [136,139,162,163,164]. Clinically relevant repeat-dose schedules should therefore include a recovery period and assess local histopathology, infection/fibrosis, organ toxicity, immune responses, systemic distribution, and delayed tumor recurrence.

### 5.3. Manufacturing, Standardization, and Scale-Up

If the proposed microneedle–sEV system advances, manufacturing should control source material, culture/harvest, sEV isolation, loading, microneedle fabrication/crosslinking, sterilization, storage, and release [19,158,159,160]. Critical process parameters should be linked to critical quality attributes spanning sEV identity/purity/potency, device performance, sterility, stability, and dose uniformity. This integrated approach is necessary because sEV and device attributes are often evaluated separately in component studies.

Scale-up, cell-bank changes, reformulation, sterilization, packaging, or storage changes should trigger predefined comparability testing rather than assumed equivalence [158,159,161,184]. A qualified reference lot and acceptance ranges for potency, oncologic safety, and device performance can help distinguish genuine product consistency from similarity in particle count or marker expression alone.

Release testing should cover four domains:

- Identity/purity: particle size/concentration, morphology, markers, and contaminants [19,147,158,159,161,185,186,187,188,189,190,191,192,193,194,195].
- Product safety: sterility, endotoxin, and residual process reagents.
- Device performance: loading/dose uniformity, release, strength, insertion, and oral stability.
- Biological potency: a mechanism-linked assay on the released sEV product.

Functional consistency cannot be inferred from particle counts or marker panels alone; reference lots, predefined acceptance ranges, and potency/oncologic-safety criteria should support batch release [161,196].

### 5.4. Integration, Usability, and Future Translation

Potential clinical integration should be evaluated only after preclinical product definition. The studies of microneedle–sEV systems identified by the review remain preclinical [27,28,66,120,124,197], as do most broader nanotechnology-based combinations [198]. Future OSCC studies should define dose, sequence, cycle length, and efficacy endpoints within clinically relevant postoperative, checkpoint-therapy, and chemoradiotherapy pathways [199].

Practical development must also address posterior-site placement, wet or fragile mucosa, accidental detachment/swallowing, interference with speech/eating, and reproducible self- or clinician-administration [103,130,140,200,201,202,203,204,205,206]. In parallel, cell-source variability, two-dimensional culture inefficiency, scale-up cost, cold-chain dependence, freeze–thaw damage, and inconsistent potency assays remain manufacturing constraints [207,208,209,210,211,212,213,214,215,216]. These issues are retained here once rather than repeated in each application section.

Any future translation would require GMP-compatible sEV production, aseptic device manufacture, and a comparability plan linking process changes to critical quality attributes, potency, oncologic safety, and device performance [158,159,160,161,184,217,218].

Future diagnostic/theranostic extension. Salivary non-coding RNAs (ncRNAs) have been investigated as non-invasive OSCC biomarkers across miRNA, lncRNA, and circRNA classes [219,220]. Original studies have directly demonstrated increased salivary miR-31 in oral carcinoma [221] and diagnostic potential of salivary hsa_circ_0001874 and hsa_circ_0001971 in OSCC [222]. More specifically for the sEV rationale, salivary extracellular-vesicle-associated miRNAs have been profiled in OSCC [223], and salivary sEV-associated miR-1307-5p has been associated with disease aggressiveness and poor prognosis [224]. These findings provide a biological rationale for exploring microneedle-assisted collection of lesion-proximal sEVs as a future sampling strategy that could, in principle, enrich local molecular information and be paired with local treatment. Hydrogel microneedles have already been used to extract exosomes for early cancer detection in a colorectal-cancer model [225]. However, these studies do not establish that all diagnostically informative salivary ncRNAs are sEV-encapsulated, and microneedle-mediated enrichment of tumor-derived salivary sEVs has not been established. Any integrated theranostic application should therefore remain conceptual until lesion-specific sEV recovery, ncRNA integrity, analytical reproducibility, diagnostic/prognostic performance, and compatibility with therapeutic delivery are directly validated.

**Table 4:** Consolidated preclinical development bottlenecks for the proposed microneedle–sEV system in OSCC.

Bottleneck	Main Issue	Evidence Status	Priority Action	Refs.
Product heterogeneity	Source/process variability Batch variability	Cell state/isolation alter cargo and potency Particle markers ≠ functional equivalence	Qualify source/cell bank Lock process Define subtype + potency assay	[19,158,159]
Oncologic safety	Source-dependent pro-tumor risk Harmful cargo transfer	OSCC studies show angiogenic, immunosuppressive, EMT, and resistance pathways.	Negative oncologic-safety panel Batch stop/release criteria	[82,84,85,86,88,89]
Mechanistic evidence	Oral fate/uptake not quantified	General uptake known MN in vivo tracking absent	Quantitative tracing Spatial/omics linkage to mechanism	[181,182,183]
Quality control/consistency	No integrated CQA/CPP framework Comparability undefined	sEV and device attributes are often assessed separately.	Integrated release panel Reference lot + comparability plan	[19,158,159,160,161,184]
Long-term/repeated-dose safety	Repeated-dose oral safety unknown Delayed oncologic risk	Short-term oral/skin and route-specific EV data	Repeat-dose + recovery Large-animal oral safety Delayed recurrence	[82,84,85,86,88,89,136,139,162,163,164]
Future translation	Integration, dose, and schedule undefined	The proposed microneedle–sEV system remains preclinical.	Prospective MDT-aligned combination studies	[66,199]

## 6. Conclusions and Outlook

The proposed microneedle–sEV system remains at the preclinical conceptual stage for localized adjunctive treatment of established OSCC. Its rationale is strongest where OSCC-specific sEV mechanisms can be paired with oral-microneedle engineering, but development should proceed only through comparator-based testing of local dose, mechanism-linked potency, oncologic safety, manufacturing consistency, biodistribution, and repeated-dose oral safety. This framework is intended to guide preclinical decisions rather than imply therapeutic or clinical maturity.

Priority studies are therefore limited to four decisions:

- Source: Qualify candidate sEV sources and exclude OSCC-relevant pro-tumor activity.
- Product: Define critical process/quality attributes and mechanism-linked release assays.
- Safety: Quantify biodistribution and repeated-dose oral safety in relevant models.
- Performance: Compare the proposed microneedle–sEV system with free sEVs, blank microneedles, established local delivery, and standard therapy.

Only candidates meeting these preclinical requirements should be considered for later translational development.

A separate future extension is lesion-proximal salivary sEV sampling for ncRNA profiling; this diagnostic concept should be validated independently and does not alter the treatment-focused scope of the present review [219,220,221,222,223,224].

## Figures and Tables

**Table 3:** Candidate technical design options for the proposed microneedle–sEV system in OSCC.

Domain	Option	Advantages	Main Limitation	Candidate Design	Proposed Use	Refs.
Matrix materials	Hyaluronic acid (HA)	Biocompatible Swelling in wet media	Limited strength Weak mucosal adhesion	Blend with chitosan/other polymers.	Short-course local adjunct	[130,131]
	Chitosan (CS)	Mucoadhesive Antibacterial	Poor solubility Lower sEV loading	Genipin crosslinking Optimize molding	Postoperative wounds	[119,132]
	Silk fibroin (SF)	High strength Controlled degradation	Adhesion requires optimization.	Blend with chitosan/alginate	Sustained delivery	[128,133]
sEV loading	Matrix embedding	High/uniform loading Compatible freeze-drying	Burst release Processing damage	Trehalose/mannitol Controlled drying	Dissolving/hydrogel MNs	[144]
	Physical adsorption	Mild processing Preserved membrane	Weak binding Limited loading	Optimize surface interactions	Coated MNs	[145]
	Chemical immobilization	Strong retention Controlled release	Surface-protein modification	Genipin; control pH/temperature	Dissolving MNs	[144]
Release kinetics	Sustained release	Continuous local exposure	Degradation must match dosing.	Tune SF crystallinity/polymer ratio	Proposed postoperative use	[125,126]
	Pulsed/responsive release	Treatment-synchronized Lesion-responsive	Response specificity must be optimized.	pH-responsive or MMP-cleavable systems	Chemoradiotherapy sensitization	[127,152]
Targeting	Physical targeting	Simple No chemical modification	Placement-dependent	Direct lesion placement Flexible backing	Accessible lesions	[152,153]
	Molecular targeting	Higher tumor-specific uptake	Complex modification sEV properties may be altered.	EGFR peptides or iRGD	High-specificity targeting	[154,155]

## Data Availability

No new data were created or analyzed in this study. Data sharing is not applicable to this article.
